# Evidence-Informed Policy-Making: Are We Doing Enough? Comment on "Examining and Contextualizing Approaches to Establish Policy Support Organizations – A Mixed Method Study"

**DOI:** 10.34172/ijhpm.2022.7010

**Published:** 2022-03-07

**Authors:** Moriah E. Ellen, Eliana Ben-Sheleg

**Affiliations:** ^1^Department of Health Policy and Management, Guilford Glazer Faculty of Business and Management and Faculty of Health Sciences, Ben-Gurion University of the Negev, Be’er Sheva, Israel.; ^2^Israel Implementation Science and Policy Engagement Centre, Ben-Gurion University of the Negev, Be’er Sheva, Israel.; ^3^Institute of Health Policy Management and Evaluation, Dalla Lana School of Public Health, University of Toronto, Toronto, ON, Canada; ^4^Department of Epidemiology, Biostatistics and Community Health Sciences, Faculty of Health Sciences, Ben-Gurion University of the Negev, Be’er Sheva, Israel.

**Keywords:** Evidence-Informed Policy-Making, Knowledge Translation, Health Policy

## Abstract

In their study of manifestations of policy support organizations (PSOs), Al Sabahi et al found that PSOs are united in their goal to support evidence-informed policy-making (EIPM), albeit with differing approaches. Their article is an important contribution to the body of research on evidence utilization and implementation. The unprecedented evidence climate presented by coronavirus disease 2019 (COVID-19) provides a unique window to motivate EIPM implementation. Research such as Al Sabahi and colleagues must prompt a dialogue regarding how best to address some of the current shortcomings in the field of EIPM. Monitoring and evaluation of best practices in EIPM is scarce. EIPM uptake is unsatisfactory, and the scientific community needs to ask itself why that is and what can be done. And, we should strive to develop a gradient that discerns between the convenient and the essential so countries can evaluate and pursue the policies to best address their greatest pain points through evidence.

 In their recent article, Al Sabahi et al explore the views, experiences, and objectives of policy support organizations (PSOs) across the World Health Organization (WHO) regions. While they found that PSOs are all united in their goal to support evidence-informed policy-making (EIPM), the approaches to achieve this goal varied by organization.^1^

 Their article contributes to the way we understand the implementation of evidence utilization. Conducting policy-relevant research, packaging the evidence in appropriate formats, encouraging an accepting climate for research use, and implementing brokering mechanisms, such as PSOs that are described in the original article, are all examples of ways to support EIPM further. While the findings of this article are extremely important, we need to sometimes take a broader perspective and ask how we can push the field of EIPM to the next level. We need to ask: what more can we do to encourage EIPM uptake? How can we improve accepted practices for the evaluation of initiatives that support EIPM? And most pressingly, how do we advance evidence-informed policies beyond where they are convenient and relatively painless to where they are crucially and urgently needed?

## Monitoring, Evaluation, and Beyond

 Among their findings, the authors concluded that the most common activities that PSOs undertake to pursue policy development include supporting learning about how to make evidence-informed decisions and synthesizing research evidence. It could be that these findings are a result of the types of approaches that have been encouraged heretofore. Synthesis of research evidence and methodologies are well established. However, we lack adequate monitoring and evaluation of current EIPM practices to know and fully understand what practices work and why. Despite this, we keep pushing forward with the currently accepted practices. Since there is so much to gain from improving and expanding the reach of EIPM, it is important to reconsider the most effective techniques for monitoring and evaluation and how best to improve these processes.

 In the past two decades, there has been an explosion of interest in EIPM. It started with an awareness that patients were receiving treatments of little-known effectiveness, and the push for evidence-based medicine was born in the late 1990s. Since then, we have seen huge interest and advancement in the field of evidence-based, which later became known as evidence-informed, policy. Scientists in the field of EIPM are studying and promoting different approaches to getting evidence into action, yet the evaluations of the approaches are falling short. It is reminiscent of the adage that the ‘shoemakers’ son always goes barefoot. Academics and scientists in EIPM are doing an excellent job of promoting evidence-informed decisions. However, the methods themselves are not 100% evidence-based and not necessarily fully evaluated. While there are challenges in evaluating and monitoring methods to promote EIPM, the reality is that evaluative tools do exist. WHO EVIPnet developed a series of questions to monitor and evaluate existing programs that ask whether a given program meets current needs, whether it produces the expected results, and whether it contributes to higher-level objectives.^2^ Al Sabahi and colleagues’ article is a good evaluation of PSOs, and similar, additional work is necessary for the field of evidence implementation. However, while it is essential to increase our efforts to monitor and evaluate EIPM, it is these authors’ opinion that there is another component of EIPM that needs to be addressed, namely: are we doing enough? Perhaps the time has come to expand beyond evidence briefs and dialogues and implement more innovative approaches with which to encourage EIPM.

## Evidence-Informed Policy-Making’s Unfulfilled Potential

 The EIPM crisis is two-fold. For progress to be achieved, we must expand our understanding of the structures and issues surrounding the implementation of evidence utilization and simultaneously explore how to promote its uptake in the first place. Al Sabahi and colleagues’ article makes an important contribution to the understanding of brokering organizations. Yet, despite all the existing knowledge and frameworks, EIPM uptake does not correspond with its importance, cost benefit, and potential benefits it can serve to patients’ health. EIPM and its value have been well established for over thirty years. Despite this, the reality is that EIPM has not been embraced as much as it should be. The pressing question that we need to ask ourselves as a community of researchers is why? And how can we advance this work so it will be integrated better? Al Sabahi and colleagues’ article, while furthering the understanding of current practices, stops short of addressing how best to challenge the current unsatisfactory reality.

 The coronavirus disease 2019 (COVID-19) pandemic has afforded us a rare opportunity. We have seen science and policy developed and implemented at previously unimaginable rates. As scholars of health policy, we should examine how various countries have made use of scientists and local science while managing the pandemic. As John Lavis commented in his 2021 WHO Global Evidence-to-Policy Summit podcast, due to COVID-19 and the increased interest in evidence it has inspired, we are experiencing “a once in a generation opportunity to build or rebuild evidence support systems.” The question is, how do we leverage this unprecedented opportunity? While these authors do not have the answers, some potential suggestions could be to look at other fields of expertise and see how they have succeeded. What have been the successes in fields such as behavioural economics, psychology, marketing, and the like, in getting individuals, organizations, and systems to change their behaviours and practices? We do not need to reinvent the wheel. We need to look outside of our EIPM world and outside of our healthcare bubble to learn from excellent practices in other disciplines. We should consider including these disciplines in PSOs. One thing to keep in mind, though, is that we cannot tackle this issue in every healthcare issue and policy. We need to be strategic.

## Developing a Gradient: The Call for Evidence Where It Is Needed Most

 In a perfect world, all policies would be evidence-informed. However, the reality is that the barriers are endless, and to a large extent, policy-making lies beyond the control of policy-makers.^3^ Political and economic factors, personal values and beliefs, culture, and practical barriers all stand in the way of EIPM.^4,5^ Due to the great challenges we face when implementing EIPM, we must ask ourselves which battles are most pressing to fight: those that are easy to win or those causing the most pain?

 In John Kotter’s famous theory of change, he posits that to create change, we need to continuously generate short-term wins to justify our efforts and motivate continued success.^6^ But when it comes to EIPM, are short-term wins enough? Or do we want to strive first and foremost to create EIPM in our most difficult, pressing issues?

 Despite the impulse to push for evidence in all areas of health policy, it is prudent to consider that not all health issues are equally urgent, and not all policies are equally essential. When encouraging the uptake of EIPM, we need to develop a gradient. While some health concerns would be nice to solve through evidence-informed policy, others are simply paramount. Family violence, mental health, and managing a pandemic (to name a few) are issues that pose significant threats to our personal health and the health of our communities. When it comes to developing and implementing policy that is informed by evidence, these are issues on which we cannot afford to compromise. But how exactly do we ensure that our EIPM expands past the “merely beneficial” to undertake these (and other) paramount issues?

 Each country has its own burning issues, and therefore the gradient of issues should and will vary from country to country. For EIPM to be relevant and beneficial, it is crucial for communities of experts to decide on the top 10% of issues in their context, country, and or organization. Each country has its own interest, ideas, and institutions that they need to identify and, based on those values, decide which areas of policy are the most important for EIPM to occur.^7^ The PSOs should focus on those areas. While it would be utopian to have EIPM in all areas of policy, and while the ‘quick wins’ are great, they do not always have the biggest impact or make the greatest change. There are pre-existing techniques that can assist in this process. For example, priority setting processes is a technique in which health options and alternatives are ranked systematically and explicitly according to a set of rules to prioritize health choices amidst scarce resources.^8^ Another technique is programme budgeting and marginal analyses, which lets decision-makers maximize the impact of available healthcare resources that most benefit the needs of their community.^9^ Such tools can help evaluate and pursue areas of policy that are most pressing and address the health issues that most significantly stand to benefit from policy rooted in evidence.

## An Eye to the Future

 By prioritizing top health priorities, each region can focus its energy and resources on implementing research where it is most relevant. Some are leading this charge, such as the Global Commission on Evidence’s initiative to produce a report containing recommendations regarding how to better disseminate evidence to decision-makers in everyday policy-making as well as in times of crisis (Global Commission on Evidence to Address Societal Challenges, 2021). Over the next decade, we must turn our focus to the areas in which policy-makers should work with academics to address the most burning issues. Likelihood of uptake should be in the areas of biggest pain, both financially and regarding human life. When it comes to health policy, going forward, it is imperative that we focus our efforts on those pain points rather than casting our net too wide. Yet we also should evaluate our initiatives that are meant to support EIPM. Only then can we truly understand which efforts are worth re-investing in and utilizing. We can only hope that as we succeed in addressing the critical issues of our day with relevant, constructive policy that is informed by evidence, policy-makers will begin to understand the need to use evidence to address lesser pain points and gradually let evidence lead the way in all policies.

## Ethical issues

 Not applicable.

## Competing interests

 Authors declare that they have no competing interests.

## Authors’ contributions

 All authors contributed in the preparation of this manuscript.
